# Association of Socio-Demographic Factors, Sick-Leave and Health Care Patterns with the Risk of Being Granted a Disability Pension among Psychiatric Outpatients with Depression

**DOI:** 10.1371/journal.pone.0099869

**Published:** 2014-06-25

**Authors:** Ellenor Mittendorfer-Rutz, Tommi Härkänen, Jari Tiihonen, Jari Haukka

**Affiliations:** 1 Department of Clinical Neuroscience, Division of Insurance Medicine, Karolinska Institutet, Stockholm, Sweden; 2 National Institute for Health and Welfare, Helsinki, Finland; 3 Department of Clinical Neuroscience, Centre for psychiatric research, Karolinska Institutet, Stockholm, Sweden; 4 Department of Forensic Psychiatry, University of Eastern Finland, Kuopio, Finland; 5 Faculty of Medicine, Department of Public Health, University of Helsinki, Helsinki, Finland; Institute of Psychiatry, United Kingdom

## Abstract

**Background:**

Depression ranges among the leading causes of early exit from the labor market worldwide. We aimed to investigate the associations of socio-demographic factors, sickness absence, health care and prescription patterns with the risk of being granted a disability pension in psychiatric outpatients with depression.

**Methods:**

All non-retired patients aged 18–60 years and living in Sweden 31.12.2005 with at least one psychiatric outpatient care visit due to a depressive episode during 2006 (N = 18034): were followed from 01.01.2007 to 31.12.2010 with regard to granting of all-cause and diagnosis-specific disability pension. Uni- and multivariate Rate Ratios (RR) and 95% Confidence Intervals (CI) were estimated for the various risk markers by Poisson Regression.

**Results:**

During the four years of follow-up, 3044 patients (16.8%) were granted a disability pension, the majority due to mental disorders (2558, 84%). In the multivariate analyses, being female, below 25 or above 45 years of age, with low educational level, living alone, residing outside big cities and being born outside Europe were predictive of a granted disability pension. Frequent in- and outpatient care due to mental disorders, prescription of antidepressants and long sickness absence spells were also associated with an increased risk of disability pension (range of RRs 1.10 to 5.26). Somatic health care was only predictive of disability pension due to somatic disorders. The risk of being granted a disability pension remained at the same level as at the start of follow-up for about 1.5 years, when it started to decrease and to level off at about 20% of the risk at the end of follow-up.

**Conclusions:**

Identified risk markers should be considered when monitoring individuals with depression and when designing intervention programs.

## Introduction

Worldwide, depression becomes more and more frequent. By 2030, unipolar depressive disorder has been predicted to be the leading cause of DALYs (disability adjusted life years) in high-income countries [1]. In parallel to the increasing trends of depression, there was an upward trend in disability pension, especially among young people and due to depression, in many European countries and the US [2], [3], [4], [5]. This development exacerbates not only the risk of labour shortages and financial strain of the society, it may also imply economic constraints and social and health consequences for people with depression [5], [6].

One of the most powerful ways in which depression may lead to social exclusion is via its impact on occupational function [7]. Securing employment for a larger number of individuals with disabilities is an important objective of policies in many European countries [3]. Of course, a limited number of people should still be anticipated to have minor chances to function in working life and economically support themselves [3]. Social protection systems are therefore generally designed to protect people against the risks of loss of income in case of e.g. disease and injury. Social benefits like a disability pension are here important to secure income for individuals with disabilities who otherwise would face repeated frustrations in trying to establish themselves on the labour market and would suffer severe economic constraints [3]. Still, disability pension as a social insurance measure, is often granted to people with depression, which is known to be positively affected by treatment and rehabilitation efforts and is likely to worsen with inactivity [8], [9]. In order to reduce the number of depressed people with early exit from the labour market, a recent OECD report highlighted the importance of research regarding the identification of risk factors for disability pension in this group [5].

Despite considerable variation of estimates, up to 22% of patients with a depression may end up being granted a disability pension depending on the length of follow-up [10], [11], [12], [13]. Predictors which have been identified to increase the risk of disability pension include severity and duration of the underlying disorder, number of previous episodes, comorbidity with another mental disorder, particularly a mental or behaviour disorder due to use of alcohol and/or comorbidity with a somatic disorder as well as old age and low educational level [10], [11], [12]. These previous studies on risk factors for disability pension in depressed patients are often based on clinical samples restricted to relatively small sample sizes and high drop-out rates and restricted information on socio-demographics. Register studies can here overcome previous methodological shortcomings like selective loss to follow-up and provide sufficient power in order to analyse diagnosis-specific disability pension.

The aim of this cohort study was to examine the association of socio-demographic characteristics, antidepressant prescription, patterns of sickness absence and in- and outpatient diagnosis-specific health care with the risk of all-cause and diagnosis-specific disability pension among psychiatric outpatients with depression.

## Methods

### Ethical statement

The study population was based on linkage of several public national registers. Ethical vetting is always required when using register data in Sweden. The ethical vetting is performed by regional ethical review boards and the risk appraisal associated with the Law on Public Disclosure and Secrecy is done by data owners. The ethical review boards can however waive the requirement to consult the data subjects (or in case of minors/children the next of kin, careers or guardians) directly to obtain their informed consent, and will often do so if the research is supported by the ethical review board and the data has already been collected in some other context. This means that for this specific study no written informed consent was given by participants (or next of kin/caregiver in the case of children) for their clinical records to be used. Patient records/information was anonymized and de-identified prior to analysis by the authority, Statistics Sweden, which was responsible for data linkage. Researchers received de-identified data. According to these standards in Sweden this project has been evaluated and approved by the Regional Ethical Review Board of Karolinska Institutet, Stockholm, Sweden.

All individuals aged 18–60 years and living in Sweden, 31.12.2005 with psychiatric outpatient care due to a depressive episode from 1.1.2006 to 31.12.2006 were selected (N  =  23806 individuals). We excluded 4797 individuals on disability pension at baseline and 975 individuals with in- and specialised outpatient care due to schizophrenia or bipolar disorder in 2006. After these exclusions, a cohort of 18034 individuals formed the study population.

### Register linkage

Register data was obtained and merged for each individual from study entry (01.01.2006) as well as retrospectively (2001–2005) up to the end of follow-up (31.12.2010) from the following registers [14], [15], [16]: 1) Longitudinal integration database for health insurance and labour market studies (LISA) held by Statistics Sweden: including socio-demographic information on age, sex, place of residence, family situation and educational status. 2) (i) National patient register including information on date and diagnosis of in- and outpatient care, (ii) Cause of death register with data on date and cause of death, and (iii) Prescribed drug register with information on type and dosage of prescribed drugs; from the National Board of Health and Welfare. 3) Micro-data for analyses of social insurance (MiDAS) with information on date and diagnosis of sickness absence and disability pension from the Social Insurance Agency (SIA). The linkages were based on the unique de-identified personal identification numbers of all residents in Sweden.

### Diagnostic criteria

All diagnoses related to risk factors and outcomes were based on the corresponding codes of the International Classification of Diseases (ICD) version 10 (ICD-10) [17]. The definition of depression from outpatient care was based on the corresponding ICD-10 code F32 (depressive episode). Mental disorders comprised ICD 10 codes F00-F99 and somatic disorders all remaining codes. Antidepressant prescription was based on the respective code in the Anatomical Therapeutic Chemical Classification System (ATC, code N06A) [18]. Mental diagnoses were categorised according to ICD10 (F00-F99) and somatic diagnoses comprised all remaining diagnoses in ICD-10.

### Socio-demographic characteristics

Baseline socio-demographic characteristics including age, sex, country of birth, region of residence, educational level and family situation from the LISA database were measured at the end of 2005 and categorized as shown in Table 1.

**Table 1:** Descriptive statistics of 18034 non-pensioned women and men aged 18–60 years, living in Sweden, and with at least one outpatient care visit due to depression in 2006, including those with disability pension due to mental and somatic diagnoses.

Baseline characteristics	All		Disability Pension	
			mental	somatic
*Socio-demographic factors*	N	%	%	%
Sex				
Women	10686	59.2	61.6	56.2
Men	7348	40.8	38.4	43.8
*Age group, years*				
18–24	3680	20.4	13.9	6.2
25–34	4739	26.3	18.3	15.0
35–44	4767	26.4	28.5	27.8
45–54	3450	19.1	26.0	31.7
55–60	1398	7.7	13.3	19.3
*Educational level, years*				
Low (≤9)	3947	21.8	25.6	24.5
Medium (10–12)	8763	48.6	50.2	51.0
High (≥12)	5140	28.5	23.4	24.3
Missing	184	1.0	0.7	0.2
*Family situation* 1				
Married/living with partner & children	5010	27.8	29.1	36.6
Married/living with partner no children	1196	6.6	9.3	13.6
Single no children	8343	46.3	43.4	36.4
Single with children	2286	12.6	14.1	10.9
18–20 year olds living with parents	1199	6.6	4.0	2.5
*Area of residence* 2				
Big cities	7402	41.0	40.5	29.2
Medium-sized cities	6219	34.4	32.1	40.9
Small cities/villages	4413	24.5	27.4	29.8
*Country of birth*				
Sweden	14256	79.0	75.2	72.6
Other Northern European countries	492	2.7	3.3	2.9
EU25 without North. Europ countries	427	2.4	2.9	2.5
Rest of the world	2859	15.8	18.7	22.0
***Previous health care and treatment, 2001*** **–** ***2005***				
Outpatient care, mental diagnosis				
No care	10826	60.0	53.6	65.6
< = median (3 visits)	3070	17.0	21.9	20.8
> median visits	4138	23.0	24.6	13.6
Outpatient care, somatic diagnosis				
No care	2670	14.8	12.7	9.1
< = median (6 visits)	7554	41.9	45.2	38.9
> median	7810	43.3	42.1	52.1
Inpatient care, mental diagnosis				
No care	15037	83.4	79.6	89.3
< = median (13 days)	1458	8.1	8.4	5.1
> median	1539	8.5	12.0	5.6
Inpatient care, somatic diagnosis				
No care	12033	66.7	65.1	48.1
< = median (3 days)	2772	15.4	18.0	19.3
> median visits	3229	17.9	16.9	32.5
Sickness absence, days, 2005				
0	9723	53.9	30.9	25.3
1–14	680	3.8	2.3	3.7
15–90	1973	10.9	11.8	13.0
90–181	1150	6.4	9.3	8.6
−365	4508	25.0	45.8	49.4
Antidepressant prescription, 2005				
0	8853	49.1	37.8	44.0
> = 1	9181	50.9	62.2	56.0
***Ongoing health care and treatment in 2006***				
Outpatient care, mental diagnosis				
< = median (2 visits)	6538	36.3	41.4	58.0
> median visits	11496	63.7	58.6	42.0
Outpatient care, somatic diagnosis				
No care	7638	42.4	39.8	24.3
< = median (2 visits)	3651	20.2	20.6	18.5
> median	6745	37.4	39.6	57.2
Inpatient care, mental diagnosis				
No care	15524	86.1	81.4	87.4
< = median (12 days)	1237	6.9	7.3	7.2
> median	1273	7.0	11.3	5.3
Inpatient care, somatic diagnosis				
No care	15716	87.1	86.7	75.3
< = median (2 days)	936	5.2	7.3	9.3
> median visits	1382	7.7	5.9	15.4
Sickness absence, days, 2006				
0	7522	41.7	17.6	11.7
1–14	522	2.9	0.8	1.9
15–90	2197	12.2	3.8	6.0
90–181	1641	9.1	5.9	8.2
−365	6152	34.1	72.0	72.2
Antidepressant prescription, 2006				
0	2782	15.4	11.8	8.6
> = 1	15252	84.6	88.2	91.4

1 children living at home; single includes single/divorced/separated/widowed;

2 Area of residence: big cities: Stockholm, Gothenburg and Malmö; medium-sized cities: cities with more than 90 000 inhabitants within 30 km distance from the centre of the city; small cities/villages [31].

### Medical treatment

Information on in- and outpatient care due to mental and somatic diagnoses in the years prior to baseline (2001–2005) and during the year of observation, 2006, was used in the analyses and categorised based on the median, see Table 1. In addition prescriptions of antidepressants in July to December the year prior to baseline, 2005, and in the year of observation, 2006, was considered in the analyses.

### Sickness absence

All people above the age of 16, living in Sweden, with an income from work or unemployment benefits, who due to disease or injury have a reduced work capacity, are covered by the national sickness insurance and can receive sickness benefits [2]. After a first qualifying day, the employer pays sick pay for the first 14 days of a sick-leave spell, thereafter sickness benefit is paid by the Social Insurance Agency. Self-employed people have more qualifying days, and they as well as unemployed people get all sickness benefits from the Social Insurance Agency. A physician certificate is required after seven days of self-certification. Sickness absence can be graded: full-time (100%) and part-time (25, 50 and 75%). The number of sickness absence days regardless of grade is termed “gross” days.

Information on duration of sickness absence in days (“gross”) the year preceding baseline, 2005, and the year of observation, 2006, with benefits from the Social Insurance Agency was used and categorised according to Table 1.

### Measurement of outcome

Granted disability pension (full- or part-time) registered in SIA was defined as the outcome measure. From 2003 onwards, disability pension for individuals between 19 and 29 years of age can be temporarily (maximum 3 years) granted in Sweden if disease or injury has impaired the individual's work capacity or for delayed completion of upper-secondary school [2]. Disability pension can be granted permanently for individuals of 30 years and older in case of reduced work capacity.

### Statistical analysis

The association of the socio-demographic factors, diagnosis-specific in- and outpatient health care, antidepressant prescription and sickness absence duration on the disability pension risk were analyzed using the Poisson regression analysis to estimate crude and multivariate Rate Ratios (RR) with their 95% Confidence Intervals (CI) [19]. Individuals were followed up from 01.01.2007 to 31.12.2010. The date of first granting of a disability pension was considered. Censoring was due to emigration and death. The follow-up time was split into 3 month intervals for the regression analyses. Main effect models were applied. Multivariate models were analysed for all-cause and diagnosis-specific disability pension (excluding collinear variables). R software with Epi-package was used in data-analyses [20], [21].

## Results

From the 18034 psychiatric outpatients with depression, 10686 (59.2%) were women (Table 1). In the five years of follow-up, 3044 patients (16.8%) were granted a disability pension, the majority due to mental disorders (N = 2558, 84%). The predominant diagnostic groups of disability pensions due to mental disorders comprised depressive disorders (n = 1504, 59%). Musculoskeletal disorders (n = 146, 30%) represented the most frequent diagnostic group among disability pensioners due to somatic disorders. The majority of the outpatients was female (59%), below 44 years (73%), lived outside big cities (59%), was born in Sweden (79%), had accomplished more than 9 years of compulsory education (77%) and was above 20 years of age and not cohabiting with a partner (59%).

Prior to baseline, as many as 40% and 85% of these outpatients had at least one outpatient care visit due to mental and somatic diagnoses (2001-05), respectively, around half of them were sickness absent (46.1%) and/or with any antidepressant prescription (50.9%) in 2005. Also inpatient care due to mental and somatic diagnoses before baseline (2001-05) was rather common (16.6% and 33.3%, respectively), Table 1. During the year of observation, 2006, 63.7% and 37.4% of the outpatients due to depression had more than 2 outpatient care visits (median) due to mental and somatic diagnoses, respectively. In total, 14% and 13% of the study population had any inpatient care due to mental or due to somatic disorders, respectively. More than half of the patients were on sick leave, the majority more than half a year (34.1%), and an overwhelming proportion of 84.6% were prescribed antidepressants at least once during the year of observation.

Compared to the whole group of outpatients due to depressive disorders, the proportions of those who were granted a disability pension due to a mental disorder during follow-up were higher for women, for those older than 35 years, with low education, living outside big cities or being born outside Europe, as well as for those with any previous or current in- or outpatient care due to a mental or somatic disorders, any prescribed psychotropic medication or sickness absence (particularly if exceeding one year) (Table 1). The proportions of those with subsequent disability pension due to a somatic disorder differed somewhat to those with such pensions due to mental disorders: they were more often men, older, living outside big cities or being born outside Europe. While specialised mental health care was less frequent in depressed outpatients with subsequent disability pension due to somatic as compared to disability pension due to mental disorders, the opposite was true for health care due to somatic disorders.

With respect to the socio-demographic characteristics, individuals more than 35 years of age, those not having achieved a high level of education and not born in Sweden, cohabiting with a partner but without children living at home, and living in small cities or villages had an increased risk of being granted a disability pension during the four years of follow-up in the crude analyses (Table 2). Regarding health care prior to baseline, those with more than median visits/days in in- and outpatient care regardless of diagnoses, sickness absence exceeding 2 weeks and being prescribed antidepressants were at increased risk of granted disability pension. With regard to ongoing health care, depressed patients with in- and outpatient care due to both somatic and mental diagnoses had increased Rate ratios for disability pension (RRs ranging from 1.32 to 1.82). In 2006, having a prescription of antidepressants (RR 1.46; 95% CI 1.3–1.6) and being on sick leave 3 to 6 months and more than 6 months was associated with significantly increased RRs (1.68 and 6.38), respectively.

**Table 2:** Crude Rate Ratios (RR) and 95% Confidence Interval for being granted a disability pension (DP) 2007–2010, 18034 non-pensioned women and men aged 18–60 years and living in Sweden with at least one outpatient care visit due to depression in 2006.

Characteristics	DP, N	%	RR (95% CI)
***Socio-demographic factors***			
Sex			
Women	1849	17.3	1.06 (0.9–1.1)
Men	1195	16.3	1
*Age group, years*			
18–24	385	10.5	1
25–34	540	11.4	1.11 (0.9–1.3)
35–44	864	18.1	1.83 (1.6–2.1)
45–54	820	23.8	2.48 (2.2–2.8)
55–60	435	31.1	3.43 (2.9–3.9)
*Educational level, years*			
Low (≤9)	717	18.2	1.23 (1.1–1.4)
Medium (10–12)	1533	17.5	1.18 (1.1–1.3)
High (≥12)	775	15.1	1
Missing	19	10.3	0.66 (0.4–1.0)
*Family situation*			
Married/living with partner with children^1^	923	18.4	1
Married/living with partner no children^1^	305	25.5	1.45 (1.3–1.6)
Single/divorced/separated/widowed no children^1^	1288	15.4	0.82 (0.8–0.9)
Single/divorced/separated/widowed with children^1^	414	18.1	0.98 (0.9–1.1)
Adolescents living with parents, 18–20 years^1^	114	9.5	0.49 (0.4–0.6)
*Area of residence* 2			
Big cities	1177	15.9	1
Medium-sized cities	1021	16.4	1.03 (0.9–1.1)
Small cities/villages	846	19.2	1.22 (1.1–1.3)
*Country of birth*			
Sweden	2276	16.0	1
Other Northern European countries	98	19.9	1.30 (1.1–1.6)
EU25 without Northern European countries	85	19.9	1.28 (1.0–1.6)
Rest of the world	585	20.5	1.33 (1.2–1.5)
*Previous health care and treatment, 2001*–*2005*			
Outpatient care, mental diagnosis			
No care	1689	15.6	1
< = median (3 visits)	509	16.6	1.07 (0.9–1.1)
> median visits	846	20.4	1.04 (1.2–1.5)
Outpatient care, somatic diagnosis			
No care	369	13.8	1
< = median (6 visits)	1189	15.7	1.16 (1.0–1.3)
> median	1486	19.0	1.42 (1.3–1.6)
Inpatient care, mental diagnosis			
No care	2470	16.4	1
< = median (13 days)	230	15.8	0.96 (0.8–1.1)
> median	344	22.4	1.42 (1.3–1.6)
Inpatient care, somatic diagnosis			
No care	1900	15.8	1
< = median (3 days)	451	16.3	1.03 (0.9–1.1)
> median visits	693	21.5	1.41 (1.3–1.5)
Sickness absence, days, 2005			
0	913	9.4	1
1–14	75	11.0	1.18 (0.9–1.5)
15–90	366	18.6	2.06 (1.8–2.3)
90–181	278	24.2	2.79 (2.4–3.2)
−365	1412	31.3	3.89 (3.6–4.2)
Antidepressant prescription, 2005			
0	1180	13.3	1
> = 1	1864	20.3	1.59 (1.5–1.7)
***Ongoing health care and treatment in 2006***			
Outpatient care, mental diagnosis			
< = median (2 visits)	751	11.5	1
> median visits	2293	19.9	1.82 (1.7–1.9)
Outpatient care, somatic diagnosis			
No care	1136	14.9	1
< = median (2 visits)	617	16.9	1.15 (1.0–1.3)
> median	1291	19.1	1.32 (1.2–1.4)
Inpatient care, mental diagnosis			
No care	2507	16.1	1
< = median (12 days)	211	17.1	1.06 (0.9–1.2)
> median	326	25.6	1.66 (1.5–1.8)
Inpatient care, somatic diagnosis			
No care	2585	16.4	1
< = median (2 days)	155	16.6	1.01 (0.9–1.2)
> median visits	304	22.0	1.38 (1.2–1.6)
Sickness absence, days, 2006			
0	506	6.7	1
1–14	28	5.4	0.78 (0.5–1.1)
15–90	126	5.7	0.84 (0.7–1.0)
90–181	183	11.2	1.68 (1.4–1.9)
−365	2201	35.8	6.38 (5.8–7.0)
Antidepressant prescription, 2006			
0	345	13.3	1
> = 1	2699	20.3	1.46 (1.3–1.6)

1 children living at home;

2 Area of residence: big cities: Stockholm, Gothenburg and Malmö; medium-sized cities: cities with more than 90 000 inhabitants within 30 km distance from the centre of the city; small cities/villages [32].

**Figure 1:**
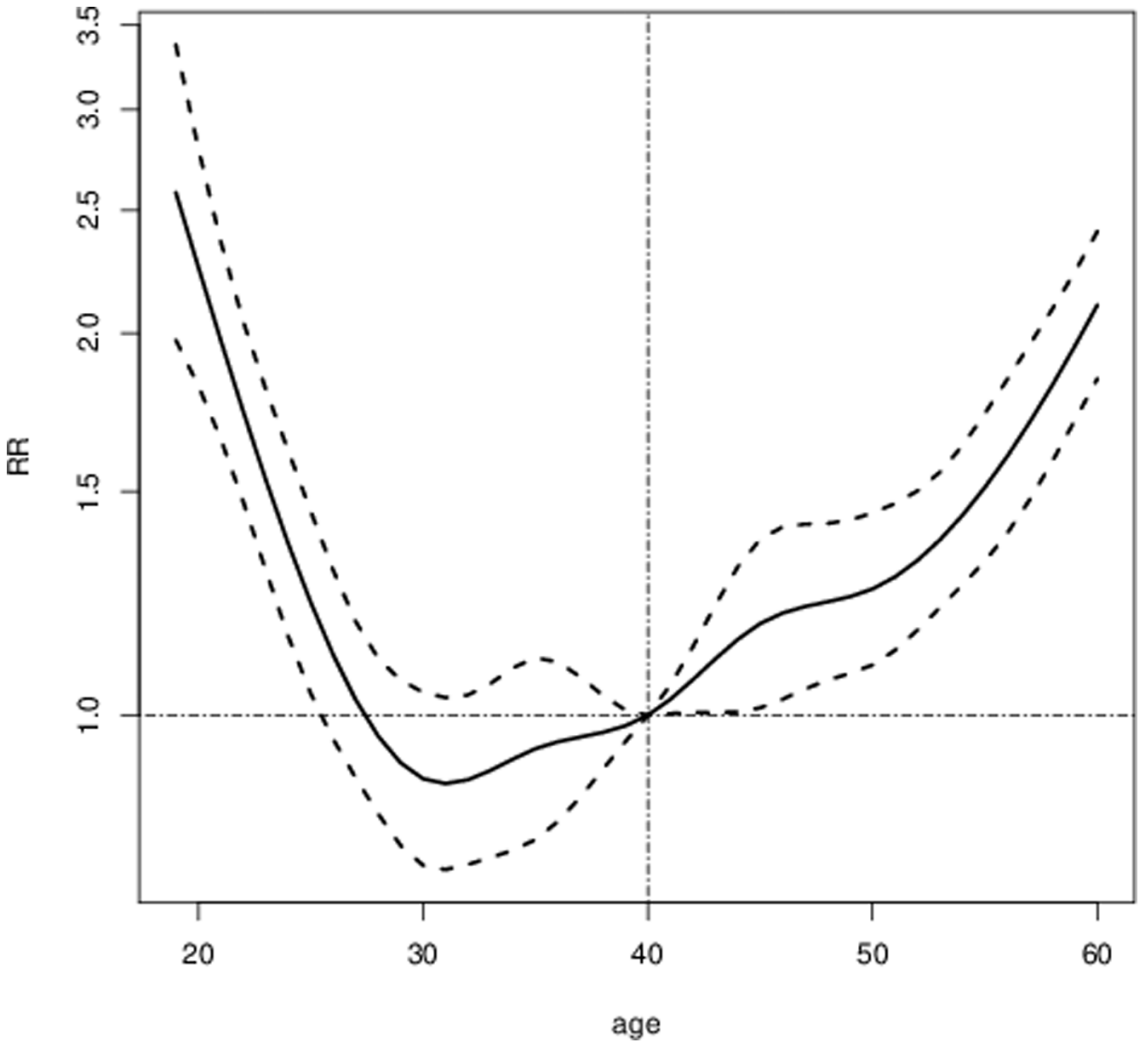
Multivariate Risk ratios (RR) and 95% Confidence intervals for the risk of being granted a disability pension across age, Reference 40 years of age. The dotted line represents the 95% Confidence Intervals.

In the multivariate analyses, being female and not having advanced to a high educational level was associated with an increased risk of being granted a disability pension in the four years of follow-up (Table 3). The association of age with the risk of granted disability pension turned out to be u-shaped (Figure 1). Being single without children living at home and not living in big cities was also associated with increased RRs for disability pension (RRs ranging from 1.13 to 1.31). The RR and 95% CI for being born outside Europe compared to being born in Sweden was 1.48 (1.3–1.6) (Table 3). Some differences in risk estimates could be observed with repect to disability pension due to mental and somatic disorders. Being female was only a risk factor for disability pension due to mental disorders, while older age and living outside big cities was more strongly associated with disability pension due to somatic disorders (Table 3).

**Table 3:** Multivariate adjusted Rate Ratios (RR) and 95% Confidence Interval for being granted a disability pension (DP) 2007–2010 due to any cause, due to mental (n = 2558) and somatic causes (n = 486), 18034 non-pensioned women and men aged 18–60 years and living in Sweden with at least one outpatient care visit due to depression in 2006.^1^

Characteristics	DP all	DP mental	DP somatic
***Socio-demographic factors***			
Sex			
Women	1.09 (1.0–1.2)	1.16 (1.0–1.2)	0.92 (0.8–1.1)
Men	1	1	1
*Age group, years*			
18–24	1	1	1
25–34	0.74 (0.6–0.8)	0.68 (0.6–0.8)	1.43 (0.8–2.4)
35–44	0.98 (0.8–1.1)	0.89 (0.8–1.0)	2.14 (1.3–3.6)
45–54	1.29 (1.1–1.5)	1.14 (0.9–1.3)	3.38 (2.0–5.6)
55–60	1.76 (1.5–2.1)	1.52 (1.3–1.8)	4.96 (2.9–8.4)
*Educational level, years*			
Low (≤9)	1.46 (1.3–1.6)	1.44 (1.3–1.6)	1.64 (1.3–2.1)
Medium (10–12)	1.18 (1.1–1.3)	1.18 (1.1–1.3)	1.23 (0.9–4.6)
High (≥12)	1	1	1
Missing	1.39 (0.8–2.2)	1.49 (0.9–2.4)	0.63 (0.8–4.9)
*Family situation* 1			
Married/living with partner with children	1	1	1
Married/living with partner no children	1.06 (0.9–1.3)	1.05 (0.9–1.2)	1.07 (0.8–1.5)
Single no children	1.12 (1.0–1.2)	1.16 (1.0–1.3)	0.99 (0.8–1.2)
Single with children	0.94 (0.8–1.1)	1.00 (0.9–1.1)	0.68 (0.5–0.9)
Adolescents living with parents, 18–20 years	1.28 (1.0–1.6)	1.24 (0.9–1.6)	1.97 (0.9–4.3)
*Area of residence* 2			
Big cities	1	1	1
Medium-sized cities	1.14 (1.0–1.2)	1.02 (0.9–1.1)	2.06 (1.6–2.6)
Small cities/villages	1.32 (1.2–1.4)	1.23 (1.1–1.4)	1.95 (1.5–2.5)
*Country of birth*			
Sweden	1	1	1
Other Northern European countries	1.10 (0.9–1.4)	1.15 (0.9–1.4)	0.87 (0.5–1.5)
EU25 without Northern European countries	1.09 (0.9–1.4)	1.13 (0.9–1.4)	0.91 (0.5–1.6)
Rest of the world	1.49 (1.4–1.6)	1.47 (1.3–1.6)	1.66 (1.3–2.1)
***Previous health care and treatment, 2001***–***2005***			
Outpatient care, mental diagnosis			
No care	1	1	1
< = median (3 visits)	0.94 (0.9–1.0)	0.94 (0.9–1.1)	0.80 (0.6–1.0)
> median visits	1.00 (0.9–1.1)	1.10 (0.9–1.2)	0.55 (0.4–0.7)
Outpatient care, somatic diagnosis			
No care	1	1	1
< = median (6 visits)	1.08 (0.9–1.2)	1.06 (0.9–1.2)	1.16 (0.8–1.6)
> median	1.13 (0.9–1.3)	1.10 (0.9–1.3)	1.36 (0.9–1.9)
Inpatient care, mental diagnosis			
No care	1	1	1
< = median (13 days)	0.86 (0.8–0.9)	0.86 (0.8–0.9)	0.55 (0.4–0.8)
> median	0.94 (0.8–1.1)	0.94 (0.8–1.1)	0.55 (0.4–0.8)
Inpatient care, somatic diagnosis			
No care	1	1	1
< = median (3 days)	0.91 (0.8–1.0)	0.87 (0.8–0.9)	1.20 (0.9–1.5)
> median visits	1.12 (1.0–1.3)	0.98 (0.9–1.1)	1.90 (1.51–2.4)
Antidepressant prescription, 2005			
0	1	1	1
> = 1	1.09 (1.0–1.2)	1.13 (1.0–1.2)	0.96 (0.8–1.2)
***Ongoing health care and treatment in 2006***			
Outpatient care, mental diagnosis			
< = median (2 visits)	1	1	1
> median visits	1.28 (1.2–1.4)	1.39 (1.3–1.5)	0.89 (0.7–1.1)
Outpatient care, somatic diagnosis			
No care	1	1	1
< = median (2 visits)	1.05 (0.9–1.2)	1.02 (0.9–1.1)	1.35 (1.0–1.8)
> median	1.03 (0.9–1.1)	0.94 (0.9–1.0)	1.77 (1.4–2.3)
Inpatient care, mental diagnosis			
No care	1	1	1
< = median (12 days)	1.08 (0.9–1.2)	1.12 (0.9–1.3)	0.94 (0.7–1.3)
> median	1.22 (1.1–1.4)	1.35 (1.2–1.5)	0.63 (0.4–0.9)
Inpatient care, somatic diagnosis			
No care	1	1	1
< = median (2 days)	0.95 (0.8–1.1)	0.92 (0.8–1.1)	1.08 (0.8–1.5)
> median visits	1.08 (0.9–1.2)	0.91 (0.8–1.1)	1.65 (1.3–2.2)
Sickness absence, days, 2006			
0	1	1	1
1–14	0.79 (0.5–1.2)	0.66 (0.4–1.0)	1.59 (0.8–3.2)
15–90	0.86 (0.7–1.0)	0.79 (0.6–0.9)	1.32 (0.8–2.1)
90–181	1.51 (1.3–1.8)	1.43 (1.2–1.7)	2.07 (1.3–3.2)
−365	5.28 (4.6–6.0)	5.27 (4.6–6.1)	5.53 (3.9–7.8)
Antidepressant prescription, 2006			
0	1	1	1
> = 1	1.17 (1.0–1.3)	1.10 (0.9–1.2)	1.64 (1.2–2.3)

1 children living at home and single stands for single/divorced/separated/widowed;

2 Area of residence: big cities: Stockholm, Gothenburg and Malmö; medium-sized cities: cities with more than 90 000 inhabitants within 30 km distance from the centre of the city; small cities/villages [32].

**Figure 2:**
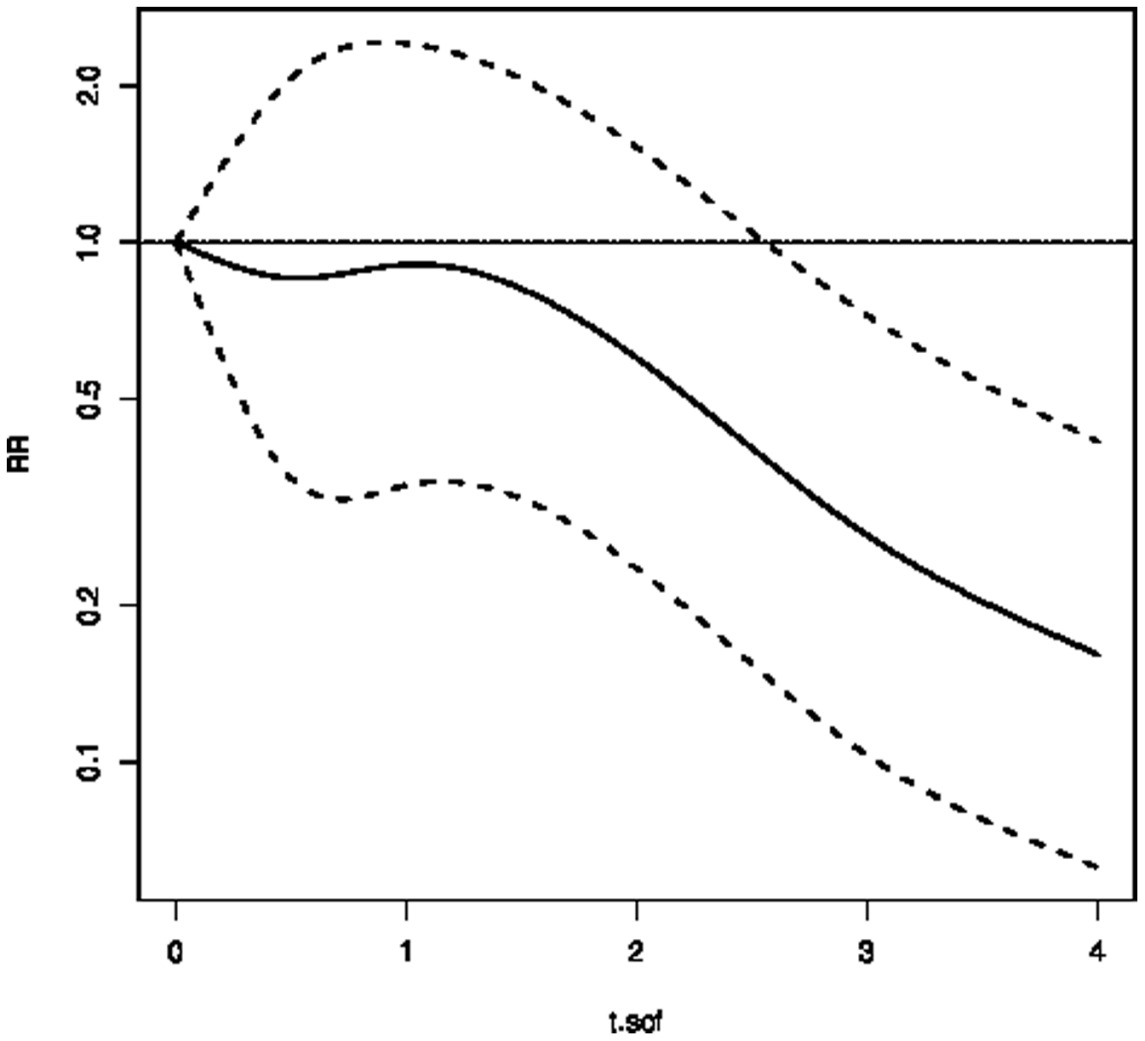
Multivariate Rate ratios (RR) and 95% Confidence intervals for the risk of being granted a disability pension over the four years of follow-up, Reference: start of follow-up at 01.01.2007. t.sof stands for time since start of follow-up in years.

With respect to ongoing health care treatment, the risk for disability pension in the multivariate analyses was increased for more than median days/visits in in- and outpatient care due to mental disorders as well as for antidepressant prescription in 2006 (RRs ranging from 1.18 to 1.31). Also sickness absence between 3 and 6 months (RR 1.56, 95% CI 1.3–1.9) and exceeding 6 months (RR 5.26, 95% CI 4.6–5.9) was predictive of granted disability pension (Table 3). Sickness absence below three months turned out to be protective for disability pension due to mental disorders. Previous and current health care due to somatic disorders was only predictive for disability pension due to somatic disorders. The risk of being granted a disability pension remained at the same level as at the start of follow-up for about 1.5 years, when it started to decrease and to level off at about 20% the risk at the end of follow-up (Figure 2).

## Discussion

### Main findings

In the multivariate analyses, being female, below 25 or above 45 years of age, with low educational level, living alone and outside big cities as well as being born outside Europe was predictive of being granting disability pension. Ongoing episodes of mental disorder necessitating more than the median days/visits in in- or outpatient care or prescription of antidepressants as well as long duration of sickness absence exceeding three months were also associated with an increased risk of disability pension. Some differences were observed with regard to risk estimates related to disability pension due to mental as compared to somatic disorders.

### Sex

We found that women had a slightly increased risk of being granted a disability pension due to any cause and due to mental disorders during the four years of follow-up compared to their male counterparts. This is in line with a previous study [13]. It is noteworthy that this increased risk remained after control for a number of socio-demographic factors and previous and ongoing diagnosis-specific health care, sickness absence and prescription of antidepressants and that there was no excess risk with regard to disability pension due to somatic disorders.

### Age

In the multivariate analyses, a u-shaped association of age with the risk of granting disability pension, particularly due to mental disorders, was found. This contrasts previous studies reporting a significantly increased risk of disability pension with increasing age [11], [12], [13]. A linear increasing trend with age was in our study only found for disability pension due to somatic disorders. An explanation that not only individuals above 55 years of age had an increased risk of disability pension due to mental disorders, but also the youngest (18–24 years of age) might be related to changes in the social insurance system in Sweden in 2003 [2]. From 2003 and onwards, disability pension for individuals below 30 years can be temporarily (maximum 3 years) granted if disease or injury has impaired the individual's work capacity or for delayed completion of upper secondary school. These disability pensions are to more than 70% due to mental disorders [2]. Further studies are warranted to elucidate these findings, particularly as granting of a disability pension in young age, even if temporal, will make it difficult to enter the labour market at a later stage. This could in turn lead to inactivity, social isolation, a sense of failure, and a lack of meaning, particularly as the entrance into the labour force and to be able to earn one's own living is a major step in the transition into adulthood [22], [23], [24].

We found that the risk of disability pension was increased in individuals with low and medium level of education and those being born outside Europe. The inverse association of educational level and disability pension risk in depressed patients is in line with a previous study [13]. The finding that first generation immigrants have a higher risk for disability pension was earlier reported in studies based on the general population [25] and individuals on long term sick-leave [26], but to our best knowledge not on patients with depression. Differences in diagnostics and treatment in the native population compared to immigrants with a different cultural background have previously been noted and suggested to contribute to the higher risk of immigrants to become disability pensioners [27]. A previous study from the Netherlands, however, could not find an elevated risk of disability pension once controlling for occupation, income, working conditions, health and mental distress [28]. Further studies are warranted in order to elucidate mechanisms behind the higher risk of disability pension among immigrants, particularly those with a country of birth outside Europe.

Findings of this study further indicate that individuals with outpatient care due to a depressive episode, who lived outside big cities had an increased risk of being granted a disability pension, particularly due to somatic disorders, during the four years of follow-up. To the best of our knowledge, this has not been reported in studies based on depressed patients, but in studies based on the general population [29]. The associations in our study remained after adjustment of differences in in- and outpatient health care, prescriptions of antidepressants and a number of socio-demographic factors. These findings might reflect regional differences in access to health care, work-place rehabilitation and local labor market policies.

Sickness absence exceeding three months was found to be related to an increased risk of disability pension, while sickness absence below three months was not associated with an excess risk related to disability pension due to mental disorders. This finding is in line with a previous study, which however, did not include any information on the length of sickness absence [12]. While the association of sickness absences exceeding three months with disability pension may reflect the severity of the disease and the mere fact that sickness absence is a precondition of disability pension, the finding of lower risk related to shorter sickness absences (less than three months) merits further research. This association remained after controlling for differences in previous and ongoing health care and treatment.

### Ongoing health care

In the multivariate analyses, ongoing in- and outpatient health care due to somatic diagnoses, was not a significant predictor of all-cause disability pension in depressed patients. This is in contrast to previous studies reporting a significantly increased risk of disability pension in depressed patients given a comorbidity with somatic disorders [10], [13]. While in- and outpatient care due to somatic diagnoses might be a good marker for the severity of the underlying somatic disorders necessitating in- or outpatient care, information on somatic disorders not treated in specialized health care was missing and may explain the differences in findings. On the other hand, previous and current somatic health care was predictive of disability pension due to somatic disorders in depressed outpatients. Proper treatment, rehabilitation and follow-up of a comorbid somatic disorder in outpatients with a depressive disorder is warranted in order to reduce the number of these patients with early exit from the labor market.

Ongoing antidepressant prescription and in- and specialized outpatient care due to mental disorders exceeding the median number of days/visits turned out to be significant predictors of disability pension, particularly of such pension due to mental disorders. While crude measures of antidepressant treatment and frequent treatment in specialized psychiatric health care might reflect markers of more severe symptoms [12], futures studies are warranted to investigate the optimal treatment of depressed patients in order to prevent early exit from the labor market.

### Strengths and limitations

To the best of our knowledge this is the first study ever investigating the association of a wide range of socio-demographic characteristics, health care, antidepressant prescription and duration of sickness absence and the risk of being granted a disability pension based on a very large group of outpatients due to depression (exceeding 18000 patients). The main strengths of this study include the very large and population-based cohort of psychiatric outpatients, the prospective design, practically no loss to follow up, and administrative register data of good quality [14], [15], [16], which recorded exposure, confounders, and outcome independently from each other. Still, some limitations should be mentioned. The coverage of the psychiatric outpatient care register might not be 100% for all counties, still there is no reason to believe that missing information is systematically related to the outcome [30]. Second, the lack of information on shorter sick-leave spells can be regarded as both a strength and a limitation. The major part of the shorter spells is not certified by a physician, which means a lower validity. And third, only information on specialised health care was available. When using data on in- and outpatient health care, the possibility of residual confounding by unmeasured health care particularly from primary health care should be kept in mind.

The validity of disability pension diagnoses is often discussed, however, hardly studied. Nevertheless, Lungdahl et al. 1991 could show a high validity of sick-leave diagnoses compared to diagnoses from medical records [31]. In the current study disabilty pension diagnoses were grouped in broad categories of mental versus somatic disorders. Also, profound medical examinations underlying granting of a diagnosis-specific disability pension forms the basis for economic compensation - in most cases for many years, which is also in favor of a good quality of the underlying diagnoses. The information on disability pension benefits are derived from an administrative register indicating economic compensation in case of work incapacity. In general, it is understood that such economically based registers are of good quality [4].

## Conclusions

Both clinical measures reflecting the severity of the depressive disorder and an eventual comorbid disorder as well as a number of socio-demographic characteristics were predictive of disability pension, particularly in the first two years after outpatient care due to depression. Information derived from this study can contribute to the design of intervention programs in order to reduce the number of depressed patients with early exit from the labor market. These programs should consist of person-based rehabilitation efforts which take the socio-economic, ethnic and psychosocial characteristics of the patient into consideration.

## References

[1] Mathers CD, Loncar D (2006) Projections of global mortality and burden of disease from 2002 to 2030. PLoS Med 3: e442. 17132052 10.1371/journal.pmed.0030442 PMC1664601

[2] Försäkringskassan (2012) Social insurance in figures, 2012 (The Social Insurance Agency).

[3] OECD (1996) Historical statistics, Paris.

[4] Järvisalo J, Anderson B, Boedeker W, Houtman I (2005) Mental disorders as a major challenge in prevention of work disability. Helsinki: Kela.

[5] OECD (2005) Best practice for reducing sickness and disability absences. Chapter 3. Economic Survey of Sweden: Organisation for Economic Co-operation and Development.

[6] Försäkringskassan (2007:11) Analyserar, Nya skuk-och aktivitetsersättningar/förtidspensioner - med fokus på yngre med psykiska diagnoser under åren 1995–2005.

[7] Adler DA, McLaughlin TJ, Rogers WH, Chang H, Lapitsky L, et al (2006) Job performance deficits due to depression. Am J Psychiatry 163: 1569–1576. 16946182 10.1176/appi.ajp.163.9.1569 PMC4269259

[8] Kupfer DJ, Frank E, Phillips ML (2012) Major depressive disorder: new clinical, neurobiological, and treatment perspectives. Lancet 379: 1045–1055. 22189047 10.1016/S0140-6736(11)60602-8 PMC3397431

[9] Simon GE, Barber C, Birnbaum HG, Frank RG, Greenberg PE, et al (2001) Depression and work productivity: the comparative costs of treatment versus nontreatment. J Occup Environ Med 43: 2–9. 11201765 10.1097/00043764-200101000-00002

[10] Rohold A, Nielsen HL, Hasle H (1989) [Fatal thrombocytopenia triggered by quinidine]. Ugeskr Laeger 151: 2672. 2815384

[11] Sorvaniemi M, Helenius H, Salokangas RK (2003) Factors associated with being granted a pension among psychiatric outpatients with major depression. J Affect Disord 75: 43–48. 12781349 10.1016/s0165-0327(02)00034-4

[12] Rytsala HJ, Melartin TK, Leskela US, Sokero TP, Lestela-Mielonen PS, et al (2007) Predictors of long-term work disability in Major Depressive Disorder: a prospective study. Acta Psychiatr Scand 115: 206–213. 17302620 10.1111/j.1600-0447.2006.00878.x

[13] Holma IA, Holma KM, Melartin TK, Rytsala HJ, Isometsa ET (2012) A 5-year prospective study of predictors for disability pension among patients with major depressive disorder. Acta Psychiatr Scand 125: 325–334. 22054701 10.1111/j.1600-0447.2011.01785.x

[14] Ludvigsson JF, Andersson E, Ekbom A, Feychting M, Kim JL, et al (2011) External review and validation of the Swedish national inpatient register. BMC Public Health 11: 450. 21658213 10.1186/1471-2458-11-450 PMC3142234

[15] Socialstyrelsen (2009) The Cause of Death Register Available: www.socialstyrelsense.

[16] SCB (2006) (1982) (1982) Longitudinell integrationsdatabas för sjukförsäkrings- och arbetsmarknadsstudier, LISA (Longitudinal integration database for health insurance and labour market studies) (In Swedish). Statistics Sweden 4 4.

[17] Socialstyrelsen (1997) Klassifikation av sjukdomar och hälsoproblem 1997.

[18] Kessler RC, Aguilar-Gaxiola S, Alonso J, Chatterji S, Lee S, et al (2009) The global burden of mental disorders: an update from the WHO World Mental Health (WMH) surveys. Epidemiol Psichiatr Soc 18: 23–33. 19378696 10.1017/s1121189x00001421 PMC3039289

[19] Preston D (2005) Poisson regression in epidemiology. In: Armitage P, Colton T, editors. Encyclopedia of Biostatistics: Wiley, Chichester, UK. pp. 4124–4127.

[20] The R Core Team (2012) Language and Environment for Statistical Computing. Vienna, Austria. Available: http://www.R-project.org/.

[21] Bendix Carstensen (2012) Epi: A Package for Statistical Analysis in Epidemiology. R package version 1.1.40. Available: http://CRAN.R-project.org/package=Epi.

[22] Arnett JJ (2001) Conceptions of the transition to adulthood: perspectives from adolescence through midlife. J Adult Dev 8: 133–143.

[23] Waddell G, Burton K (2006) Is working good for your health and well-being? Cardiff & Huddersfield: Cardiff University & University of Huddersfield. 1–246 p.

[24] Försäkringskassan (2012) Tio år med aktivitetsersättning (Ten years with disability pension among young people.). The Swedish Social Insurance Agency, Stockholm, Sweden Dnr 06961–2011.

[25] Johansson B, Helgesson M, Lundberg I, Nordquist T, Leijon O, et al (2012) Work and health among immigrants and native Swedes 1990–2008: a register-based study on hospitalization for common potentially work-related disorders, disability pension and mortality. BMC Public Health 12: 845. 23039821 10.1186/1471-2458-12-845 PMC3532317

[26] Karlsson NE, Carstensen JM, Gjesdal S, Alexanderson KA (2008) Risk factors for disability pension in a population-based cohort of men and women on long-term sick leave in Sweden. Eur J Public Health 18: 224–231. 18245150 10.1093/eurpub/ckm128

[27] Meershoek A, Krumeich A, Vos R (2011) The construction of ethnic differences in work incapacity risks: Analysing ordering practices of physicians in the Netherlands. Soc Sci Med 72: 15–22. 21126814 10.1016/j.socscimed.2010.10.022

[28] Claussen B, Dalgard OS, Bruusgaard D (2009) Disability pensioning: can ethnic divides be explained by occupation, income, mental distress, or health? Scand J Public Health 37: 395–400. 19346283 10.1177/1403494809104220

[29] Allebeck P, Mastekaasa A (2004) Chapter 5. Risk factors for sick leave - general studies. Scand J Public Health Suppl: 49–108. 10.1080/14034950410021853 15513654

[30] Socialstyrelsen (2008) Beskrivning av vårdutnyttjande i psykiatrin - En rapport baserad på hälsodataregistren vid Socialstyrelsen (Description of health care consumption within psychiatric services - A report based on registers from the National Board of Health and Welfare).

[31] Ljungdahl LO, Bjurulf P (1991) The accordance of diagnoses in a computerized sick-leave register with doctor's certificates and medical records. Scand J Soc Med 19: 148–53.32. 1796246 10.1177/140349489101900302

[32] SCB (2003) Regionala indelningar i Sverige den 1 januari 2003. Del 1 (Regional divisions in Sweden on 1 January 2003. Part 1).

